# A survey of the educational environment for oncologists as perceived by surgical oncology professionals in India

**DOI:** 10.1186/1477-7819-10-18

**Published:** 2012-01-23

**Authors:** Chandrakanth Are, Madhuri Are, Hemanth Raj, Vijayakumar Manavalan, Lois Colburn, Hugh Stoddard

**Affiliations:** 1 Department of Surgery, University of Nebraska Medical Center, Omaha, NE; 2 Cancer Pain and Palliative Care, University of Nebraska Medical Center, Omaha, NE; 3 Cancer Institute (WIA), Chennai, Tamil Nadu, India; 4 Kidwai Memorial Institute of Oncology, Benagaluru, Karnataka, India; 5 Executive Director, Center for Continuing Education, University of Nebraska Medical Center, Omaha, NE; 6 Curriculum and Educational Research Office, University of Nebraska Medical Center, Omaha, NE

**Keywords:** Education, Oncology, perceptions, survey, India

## Abstract

**Background:**

The current educational environment may need enhancement to tackle the rising cancer burden in India. The aim of this study was to conduct a survey of Surgical Oncologists to identify their perceptions of the current state of Oncology education in India.

**Methods:**

An Institutional Review Board approved questionnaire was developed to target the audience of the 2009 annual meeting of the Indian Association of Surgical Oncology in India. The survey collected demographic information and asked respondents to provide their opinions about Oncology education in India.

**Results:**

A total of 205 out of 408 attendee's participated in the survey with a 42.7% response rate. The majority of respondents felt that Oncology education was poor to fair during medical school (75%), residency (56%) and for practicing physicians (71%). The majority of participants also felt that the quality of continuing medical education was poor and that minimal emphasis was placed on evidence based medicine.

**Conclusions:**

The results of our survey demonstrate that the majority of respondents feel that the current educational environment for Oncology in India should be enhanced. The study identified perceptions of several gaps and needs, which can be the targets for implementing measures to enhance the training of Oncology professionals.

## Background

According to the World Cancer Report released by the International Agency for Research in Cancer (IARC) in 2008, cancer has surpassed cardiovascular disease as the leading cause of death in January 2010 [1]. The report estimated that in 2008 there will be 12 million new cases, 7 million deaths and 25 million persons living with cancer within five years of diagnosis [1]. By 2030, it is estimated that there will be 26 million new cases diagnosed annually. This increase in the global cancer burden will be mainly due to a disproportionate rise of newly diagnosed cancer cases in the developing countries such as India. India, China and Russia are predicted to account for more than half (53%) of the cancer cases and 60% of the cancer-related deaths [1]. Several factors have been cited for this disproportionate rise of new cancer cases in the developing world such as, rising population [2], enhanced life span [3-5], greater tobacco consumption [6-8] and rising body mass index [9-16].

The challenge of tackling this rising cancer burden in the developing world requires an adequate number of health care professionals who are trained in Oncology. It is well known that there are fewer health care professionals who live in the developing world countries [17]. The World Health Organization (WHO) report in 2006 noted that, in absolute terms, countries like India, Bangladesh and Indonesia have the greatest shortage of health service providers [17]. It is estimated that an increase of 50% in the number of providers is needed to address the shortages in countries like India [17]. Unfortunately, the data also suggest that the existing educational system for training Oncology care providers, although currently adequate, is not capable of supporting increases of this magnitude [3]. As of 2009 there were six Surgical Oncology fellowship (Mch) programs in India that could train a total of approximately 16 candidates per year [3]. There are plans to increase this number and some increases in the number of Mch (50) and DNB (20) training positions have been noted since 2009. India was also one of the earliest countries in the world to offer specialty training in Surgical Oncology with an awarded degree. Despite this increase in the number of training positions in Surgical Oncology, the rising cancer burden suggests that an enhancement in the training and educational environment for Surgical Oncology professionals is needed in India.

To undertake such an enhancement would require the collaboration of Surgical Oncologists currently practicing in India. The study reported here was a survey of practicing Surgical Oncology professionals that was designed to: a) assess the current educational environment for Oncology in India and b) identify the perceived gaps and needs in Oncology education that need to be addressed.

## Methods

A survey instrument was designed and delivered to Surgical Oncologists who attended the annual meeting of the Indian Association of Surgical Oncology (IASO) in 2009. A review of relevant literature did not reveal any specific reports or appropriate survey instruments that would assess the educational environment for Oncologists in India. A 31 item questionnaire was developed by the authors. The questionnaire and protocol were approved by the University of Nebraska Medical Center Institutional Review Board as exempt research (IRB: 385-09-EX). The questionnaire was originally developed as a web-based form in Survey Monkey^® ^that could be accessed during the IASO 2009. Due to difficulties of internet access at the meeting venue, a printed version of the survey was also made available.

**Figure 1:**
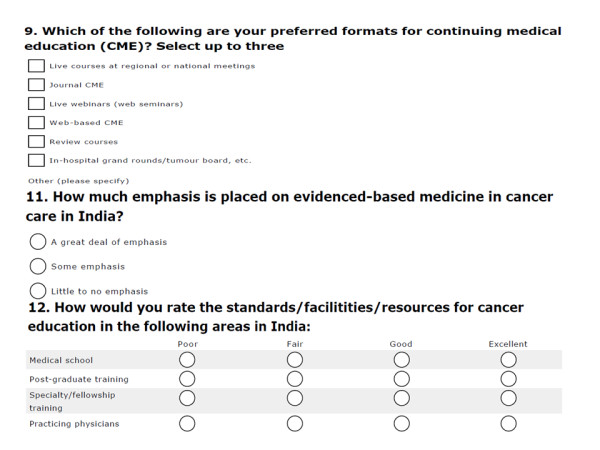
**A sample of the survey instrument**.

Participants were recruited via signage and announcements during the conference. The survey administration took place in a dedicated space in the conference facility and the authors were available to answer any questions from prospective participants about either the survey form itself or about accessing Survey Monkey^®^. Completion time ranged from 15 to 30 minutes, depending on which format of the survey was used and the time spent completing open-ended responses. Each participant received a 1 giga byte USB stick as a gift after completing the survey. Data were analyzed using SPSS (Statistical Package for the Social Sciences) v17. Descriptive analyses were done on the demographic and personal data provided by respondents and the responses to the survey items. A sample of the survey instrument is shown in Figure 1

## Results and discussion

There were a total of 220 surveys that were collected from 480 attendees at the IASO meeting (120 via Survey Monkey and 88 via print format). After reviewing the data obtained via Survey Monkey, 15 surveys were discarded due to substantial missing data resulting from internet access issues. The response rate based on the remaining 205 surveys was response rate of 42.7%.

Table 1 provides details on the demographic characteristics of those completing the survey. Over half of the respondents were younger than 40 and were within ten years of completing their initial surgical training and 92% were male. Sixty four percent of respondents indicated having completed fellowship training in Surgical Oncology. The vast majority of participants (86%) reported that they were currently practicing in an academic setting. Nearly 60% worked in a government run medical school or hospital.

**Table 1:** Demographics of survey participants

	n	% of respondents
Gender		
Male Female	186 17	91.6 8.4

Age		
29 or younger 30-39 40-49 50 and older	31 78 47 45	15.4 38.8 23.4 22.4

Time since completion of residency		
5 years or less 6 to 10 years 11 to 15 years 16 years or more	57 34 33 50	32.8 19.5 19.0 28.7

Completed Oncology Fellowship (Mch or DNB)		
Yes No	71 126	36.0 64.0

Type of Practice		
Academic Non-academic	166 27	86.0 14.0

Time spent teaching		
25% of less 26 to 50% More than 50%	49 88 30	29.3 52.7 18.0

Employment Type		
Corporate Government Private Practice	59 110 15	32.1 59.8 8.2

Mch: Magister Chirurgiae

DNB: Diplomate of National Board

### Oncology education resources

**Figure 2:**
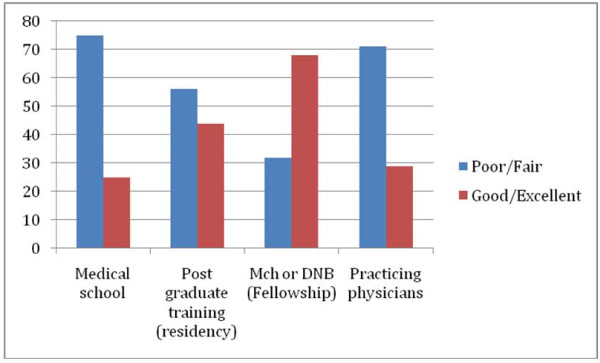
**How would you rate the standards/facilities/resources for cancer education in the following areas in India**.

The majority of respondents indicated that standards/facilities or resources for Oncology education in India were fair to poor at the undergraduate and residency education level as well as for physicians currently in practice. (Table 2 and Figure 2) Despite the aforementioned paucity of fellowship training opportunities in India, 68% of those responding indicated that the standards/facilities or resources were good or excellent at the fellowship level. The least conducive environments for cancer education were considered to be for medical students, with 75% responding fair or poor and for practicing physicians with 71% reporting fair or poor.

**Table 2:** How would you rate the standards/facilities/resources for cancer education in the following areas in India?

	n	Poor n(%)	Fair n(%)	Good n(%)	Excellent n(%)
**Medical school**	193	68 (35)	78 (40)	44 (23)	3 (2)

**Post-graduate training** **(residency)**	190	20 (10)	88 (46)	79 (42)	3 (2)

**Mch or DNB** **(Fellowship)**	186	22 (12)	37 (20)	106 (57)	21 (11)

**Practicing physicians**	175	63 (36)	62 (35)	47 (27)	3 (2)

Mch: Magister Chirurgiae

DNB: Diplomate of National Board

Those who responded either fair or poor to any of these items were also asked to write details about their response. Common themes identified among the text responses were: lack of awareness by physicians and the public about the problem, lack of a structured Continuing Medical Education (CME) system for practicing physicians, the presence of an old-style curriculum in medical school, lack of regional cancer facilities, too few training programs beyond initial residency, and lack of short-term fellowship programs.

### Participation in Continuing Medical Education (CME)

Nearly all (99%) who responded indicated that they completed some type of CME activity within the year prior to the survey. The preferred formats for CME were live courses (89%), hospital grand rounds (57%), journal CME (36%), and review courses (28%). Newer formats for CME such as internet CME and live webinars were mentioned by 14% or fewer of the respondents.

Over-two thirds of respondents indicated there were adequate opportunities for CME in India. The nearly one-third who felt there were inadequate CME opportunities indicated a wide range of issues including: the need for more local programs, poor communication of existing CME opportunities, and the poor quality of current CME programs. Comments from those responding there were few opportunities in Oncology CME indicated that there was a need for more local programs aimed at general surgeons, CME Oncology content that is specific to Oncology issues in India, as well as the awareness that Oncology as a formal specialty is still relatively new in India.

### Application of Evidence-based Medicine (EBM)

Nearly 77% of survey respondents felt that there is little-to-none or some emphasis placed on EBM in cancer care in India. Respondents attributed this to be due to a lack of resources, awareness and emphasis on EBM combined with the absence of uniformity of protocols in treatment. They also noted that the only protocols available are from outside the country and they do not seem to be appropriate for the population in India.

## Conclusions

To address the Oncology education needs of physicians in India, any changes or enhancements should start from feedback from Oncology professionals in the country. The survey of Indian Oncology professionals for the current study described the viewpoint of those professionals about the state of Oncology education in that country and generated some suggestions about means to resolve the perceived deficiencies.

The International Agency for Research in Cancer report predicted a rise in the cancer burden worldwide with a disproportionate rise in developing countries such as India [1]. It is also documented that countries like India have health care workforce shortages [3,17-19]. One avenue to address the health care workforce shortage is to educate a larger number of professionals and to expand the continuing educational opportunities for those already in practice. Most 'developing world' countries do not have the educational capacity to increase the pool of health care workers rapidly [3]. For example, although some increases in the number of fellowship training position in India has happened in the last few years, this number may still be inadequate to meet the rising burden of new cancer cases.

The ability to address the disease burden in any population depends on the availability of an adequate pool of appropriately trained health care professionals. This in turn requires the presence of a satisfactory number of training positions that will prepare those health care professionals. Beyond the training at the undergraduate, graduate and fellowship levels, there needs to be a systematic process for providing continuing medical education so that medical care adheres to the most current evidence based principles. The lack of resources for training and continuing medical education would exacerbate a shortage of health care professionals and can pose a major problem. A well-designed CME program for evidence-based practice in Oncology would contribute significantly to alleviating such shortages.

The results of our survey demonstrated that even among a group of highly specialized Oncology professionals, there was a sense that cancer education is grossly inadequate at several levels, except for residency and fellowship training. This was felt most acutely during medical school training where a large percentage (75%) felt that cancer education was poor to fair. It appears that the majority of Indian medical schools do not have separate academic divisions or student rotations dedicated to Oncology. The current curricula appear to be outdated without sufficient emphasis on how to manage the rising number of cancer cases. This is compounded by the fact there is a shortage of adequately trained faculty. Although not as acute, the survey respondents also noted that Oncology education is not ideal during post graduate training as well. The only segment of health care workers felt to have an adequate educational environment in Oncology are candidates in fellowship training. Given this and the small number of fellowship positions [3] currently available, major reforms are needed to address the problem in the near future.

The resources available to provide education for practicing physicians are also felt to be inadequate and this has been noted by other authors [20]. This is of concern since these individuals are not only the ones responsible for treating patients but also training the future pool of health care workers. Although the majority of survey respondents reported attendance at CME, many also felt there is both a shortage of CME activities and that the quality of the current activities needs to be improved. The majority of the respondents indicated they obtain their CME from attending live courses. This can be a problem in a large country such as India with limited resources and a lack of streamlined transportation facilities. There is a distinct under usage of web based educational activities which seems ironic given the perception of India as a technology center. For a country like India with a rapidly developing information technology framework, web based activities can provide a solution to address CME in remote locations.

The lack of educational curricula and CME translates into inadequate emphasis placed on evidence based practice of cancer care. Nearly four-fifths of the respondents noted that little-to-none or only some emphasis is placed on evidence based medicine. This is particularly noteworthy in cancer care where the field is in constant flux with the availability of new technology, drugs and guidelines on a regular basis. In Oncology, it is vitally important to make current treatment guidelines available to health care professionals. Lastly there are no uniform guidelines developed for the Indian population since those available from the developed world may not always be applicable to the developing world.

The results of our survey point out that, Oncology professionals perceive gaps and needs for enhanced cancer education in India. Although this study employed an exploratory survey instrument, these findings can still be of help in initiating efforts to improving Oncology education in India. Such measures may include: increasing training positions in Oncology, promote awareness of Oncology as a required specialty, updating and streamlining the existing curricula, making CME more widely available and encouraging adherence to evidence based medicine.

There are several limitations to this study. First, it represents the findings of a limited, non-representative sample. Although the meeting at which the survey was conducted was well attended and the survey response rate was adequate, additional investigation is called for before substantial investments are made. Also, although India is representative of the conditions of most developing countries, the educational context may differ in other countries even though the problems of provider shortages and inadequate training are similar in much of the developing world.

The results of this study demonstrate that there are several avenues for enhancement in Oncology education for health care professionals across the spectrum of training and practice in India. In addition, written comments from the respondents helped us to identify gaps and needs for CME. A follow-up survey administered to a representative sample in the future may be useful in targeting measures to address these gaps and needs and should help create the pool of health care professionals needed to tackle the oncoming rising burden of cancer cases in developing countries.

## List of abbreviations

Mch: Magister Chirurgiae; DNB: Diplomate of National Board

## Competing interests

The authors declare that they have no competing interests.

## Authors' contributions

CA: Design, acquisition of data, analysis and interpretation of data, drafting of manuscript, critical revision, final approval

MA: Design, drafting of manuscript, critical revision, final approval

HR: Design, acquisition of data, critical revision, final approval

MV: Design, acquisition of data, critical revision, final approval

LC: Design, acquisition of data, analysis and interpretation of data, drafting of manuscript, critical revision, final approval

HS: Critical revision, final approval

All authors read and approved the final manuscript.
